# Establishing a prognostic model of chromatin modulators and identifying potential drug candidates in renal clear cell patients

**DOI:** 10.1186/s12859-023-05229-9

**Published:** 2023-03-20

**Authors:** Puyu Liu, Jihang Luo, Na Tan, Chengfang Li, Jieyu Xu, Xiaorong Yang

**Affiliations:** 1 Department of Clinical Pathology, Affiliated Hospital of Zunyi Medical University, No.149 Dalian Road, Zunyi City, 563000 Guizhou Province China; 2 Department of Infectious Diseases, Affiliated Hospital of Zunyi Medical University, Zunyi, China

**Keywords:** Chromatin regulators, Renal clear cell carcinoma, TCGA, Prognosis model

## Abstract

**Background:**

Renal carcinoma is a common malignant tumor of the urinary system. Advanced renal carcinoma has a low 5-year survival rate and a poor prognosis. More and more studies have confirmed that chromatin regulators (CRs) can regulate the occurrence and development of cancer. This article investigates the functional and prognostic value of CRs in renal carcinoma patients.

**Methods:**

mRNA expression and clinical information were obtained from The Cancer Genome Atlas database. Univariate Cox regression analysis and LASSO regression analysis were used to select prognostic chromatin-regulated genes and use them to construct a risk model for predicting the prognosis of renal cancer. Differences in prognosis between high-risk and low-risk groups were compared using Kaplan–Meier analysis. In addition, we analyzed the relationship between chromatin regulators and tumor immune infiltration, and explored differences in drug sensitivity between risk groups.

**Results:**

We constructed a model consisting of 11 CRs to predict the prognosis of renal cancer patients. We not only successfully validated its feasibility, but also found that the 11 CR-based model was an independent prognostic factor. Functional analysis showed that CRs were mainly enriched in cancer development-related signalling pathways. We also found through the TIMER database that CR-based models were also associated with immune cell infiltration and immune checkpoints. At the same time, the genomics of drug sensitivity in cancer database was used to analyze the commonly used drugs of renal clear cell carcinoma patients. It was found that patients in the low-risk group were sensitive to medicines such as axitinib, pazopanib, sorafenib, and gemcitabine. In contrast, those in the high-risk group may be sensitive to sunitinib.

**Conclusion:**

The chromatin regulator-related prognostic model we constructed can be used to assess the prognostic risk of patients with clear cell renal cell carcinoma. The results of this study can bring new ideas for targeted therapy of clear cell renal carcinoma, helping doctors to take corresponding measures in advance for patients with different risks.

**Supplementary Information:**

The online version contains supplementary material available at 10.1186/s12859-023-05229-9.

## Introduction

The annual incidence of renal tumors accounts for 2–3% of all tumors, ranking third in urinary system tumors [1]. In 2020, through a large-scale cancer data survey of 185 countries, 431,288 patients were newly found to have kidney tumors, and 179,368 kidney cancer patients died due to the disease [2]. Renal clear cell carcinoma (KIRC) is the primary pathological type of renal tumors, accounting for about 70–80% [3]. Most renal cancer patients lack apparent symptoms and are detected incidentally through imaging examinations. If no distant metastasis occurs, the 5-year survival rate is high; however, about 30% of patients have distant metastasis at the initial assessment, and the survival rate is significantly reduced [4]. Previous studies have found that polygenic signatures provide risk stratification and prognosis prediction for cancer patients [5–8]. Therefore, this study aimed to establish a chromatin regulator signature to predict overall survival in KIRC patients. It is also used to screen for the best possible treatment drugs.

Chromatin regulators (CRs) are a class of enzymes with specialized functional domains capable of recognizing, forming and maintaining epigenetic states in a cellular context-dependent manner [9, 10]. CRs are essential upstream regulators of epigenetics. According to their regulatory roles, CRs are generally classified into three categories: histone modifications, chromatin remodelers, and DNA methylation [11, 12]. Aberrant expression of CRs is associated with various biological processes such as apoptosis, autophagy, and proliferation, suggesting that dysregulation of CRs may contribute to the development of multiple diseases, including cancer [13–15]. Therefore, CRs are expected to become new targets for treating multiple diseases. DPF3 is a component of the SWI/SNF chromatin remodelling complex. Studies have shown that DPF3a (the short isoform of DPF3) promotes renal cancer cell migration in vitro and in vivo, and the mechanism is mainly due to the specific interaction between DPF3a and SNIP1. It affects the metastasis of clear cell renal cell carcinoma (ccRCC) through the TGF-β signalling pathway [16]. The PBAF complex consists of multiple subunits, including the tumor suppressor protein PBRM1 (BAF180), which are unique to this SWI/SNF chromatin remodeling complex. PBRM1 is mutated in various cancers, with high mutation frequency in ccRCC. Deletion of PBRM1 alters promoter histone modifications and activates ALDH1A1 to drive renal cell carcinoma [17]. Some scholars obtained the expression data of KAT2A and MCT1 in RCC from The Cancer Genome Atlas (TCGA-KIRC) and the International Cancer Genome Consortium (ICGC) database. Experiments show that KAT2A is an oncogenic chromatin modifier that induces MCT1 expression to promote RCC progression, and an MCT1 inhibitor (AZD3965) can inhibit RCC [18]. Studies have also shown that BRM is a crucial subunit of the SWI/SNF chromatin remodeling complex, and the knockdown of BRM promotes the proliferation, migration and invasion of RCC cells. RGFP966 inhibits tumor progression in clear cell RCC by restoring BRM expression in vivo and in vitro [19]. HMGA1 is a chromatin remodeling factor, and studies have found that HMGA1-mediated miR-671-5p targeting APC promotes the metastasis of clear cell renal cell carcinoma through the Wnt signaling pathway [20]. The above literature show that CRs has a significant impact on the prognosis of KIRC patients, and it is worth our further consideration. However, the relationship between CRs and KIRC has rarely been systematically explored. Therefore, we used bioinformatics analysis to study the expression profile and prognostic value of CRs in KIRC. We successfully constructed a prognostic signature consisting of 11 CRs, which proved effective in predicting the prognosis of KIRC patients. In addition, we found a close relationship between this prognostic feature and immune checkpoints, which could help with immunotherapy. Based on this, we screened out 5 drugs that may be beneficial to treating KIRC patients.

## Methods

### Sources of KIRC patients and screening for differentially expressed CRs

The data analyzed in this study all come from public databases. The mRNA expression and relevant clinical information datasets were downloaded from the TCGA (https://tcga-data.nci.nih.gov/tcga) [21]. Including 541 cases of tumor tissue and 72 cases of normal tissue, and obtained the corresponding clinical data of these patients, such as age, sex, tumor differentiation degree, tumor stage, etc. Previous studies have identified 870 chromatin regulators [9] (Additional files 1, 2). Based on these CRs, we normalized the mRNA expression profiles in the KIRC data by R package (limma R package version 3.50.3). Using the limma package in R language, the criteria of |*logFC*|> 1 and false discovery rate (FDR) < 0.05 were used to identify CRs with differential expression.

### Establishment and validation of a CRs prognostic model

Univariate Cox regression analysis (survival R package version 3.4.0) was performed on differentially expressed CRs to identify genes with prognostic value. Then, a prognostic risk model was constructed by Lasso Cox regression analysis using the glmnet R package (version 4.1.4). The risk score for each sample was obtained using the following equation:

$$Risk\;score = \beta 1*Exp1 + \beta 2*Exp2 + \beta 3*Exp3 + \cdots + \beta n*Expn$$

*β* coefficient value; *Exp* gene expression level. Two subgroups (high or low risk group) were constituted based on the median risk score in these KIRC patients. Differences in overall survival (OS) time between subgroups were compared by Kaplan–Meier curves (survival R package version 3.4.0 and survminer R package version 0.4.9). In addition, the ability of the above model to predict prognosis was analyzed using the SurvivalROC R package (version 1.0.3). Randomly select 70% of KIRC TCGA cohort patients (n = 370) through the caret R package (version 6.0.93) plus patients in GSE29609 (n = 39) (https://www.ncbi.nlm.nih.gov/geo) as validation dataset to further determine whether the prognostic ability of the model was reliable.

### Nomogram establishment based on risk score and clinical variables

Univariate and multivariate Cox proportional (survival R package version 3.4.0) hazards were used to analyze risk scores and other clinical factors to determine whether their impact on prognosis was statistically significant. Based on the final model, a nomogram was constructed by the rms R package (version 6.3.0) and regplot R package (version 1.1) to predict 1, 3 and 5 years overall survival in KIRC patients. Perform a concordance index (C-index) and calibration curve to assess the predictive utility of the nomogram.

### Bioinformatics analysis

GO enrichment and KEGG (http://www.kegg.jp/kegg/kegg1.html) pathway analysis were used to analyze these differently expressed CRs. The GO analysis terms include cellular component (CC), biological process (BP), and molecular function (MF). KEGG is a comprehensive database that integrates genomic information, chemical information and biochemical system function information. All analyses were carried out by R language with org.Hs.eg.db R package version 3.14.0; clusterProfiler R package version 4.2.2; enrichplot R package version 1.14.2 and ggplot2 R package version 3.4.0. Differences were considered statistically significant when *FDR* < 0.05. Gene set enrichment analysis (GSEA) was used in different risk groups to explore the underlying molecular mechanisms. *P* value < 0.05 were considered statistically significant.

### Tumor cell immune infiltration analysis

We assessed the level of immune cell infiltration between different risk groups based on B cell-specific lncRNA signatures using the TIMER, CIBERSORT, CIBERSORT-ABS, QUANTISEQ, MCPCOUNTER, XCELL, and EPIC algorithms (pheatmap R package version 1.0.12). Immunotherapy has been proven to be an effective method for the treatment of malignant tumors. To predict which immune checkpoint inhibitors might be effective in high- and low-risk populations, we visualized differentially expressed immune checkpoints using the ggpubr R packages (version 0.4.0) [22]. In addition, this study explored the relationship between 11 CRs and immune cells through the TIMER database (https://cistrome.shinyapps.io/timer/) [23], which will help to understand the role of these CRs in the immune system of KIRC patients.

### Cancer drug sensitivity genomics analysis

The Genomics of Drug Sensitivity in Cancer (GDSC) database (https://www.cancerrxgene.org/) [24] is the largest public resource for information on drug sensitivity in cancer cells and molecular markers of drug response. To understand differences in drug sensitivity between the two risk groups, we used this database to analyze the half-maximal inhibitory concentration (IC50) of drugs. We predicted drug sensitivity by using the pRRophetic R package (version 0.5) [25].

### Statistical analysis

R software (version 4.1.3) was used for all statistical analyses in this study. Differences between the two groups were determined using the Wilcoxon test. *P* value < 0.05 were considered statistically significant.

## Results

### Prognosis-related CRs risk score model

**Fig. 1:**
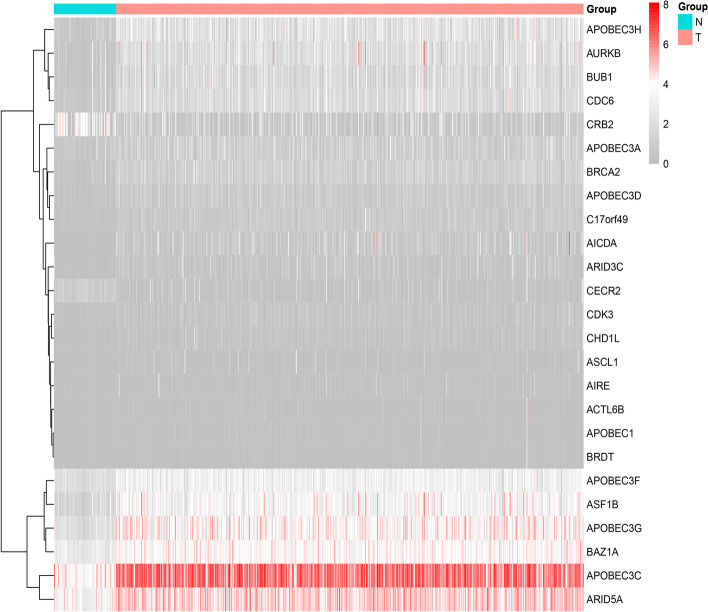
Heatmap showed TOP25 differentially expressed CRs. *N* normal, *T* tumor

**Fig. 2:**
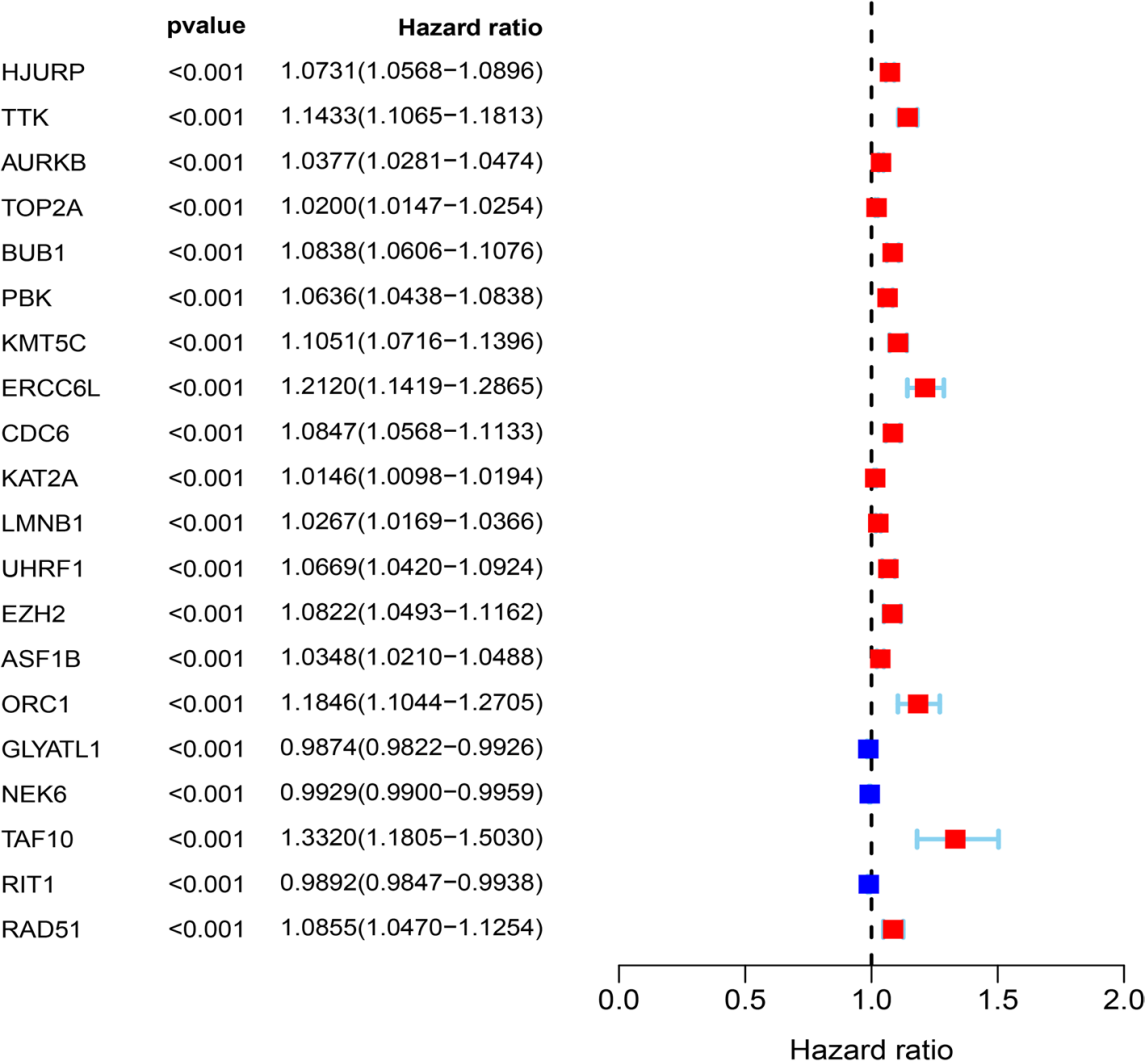
Univariate COX analysis identifies CRs associated with prognosis

**Fig. 3:**
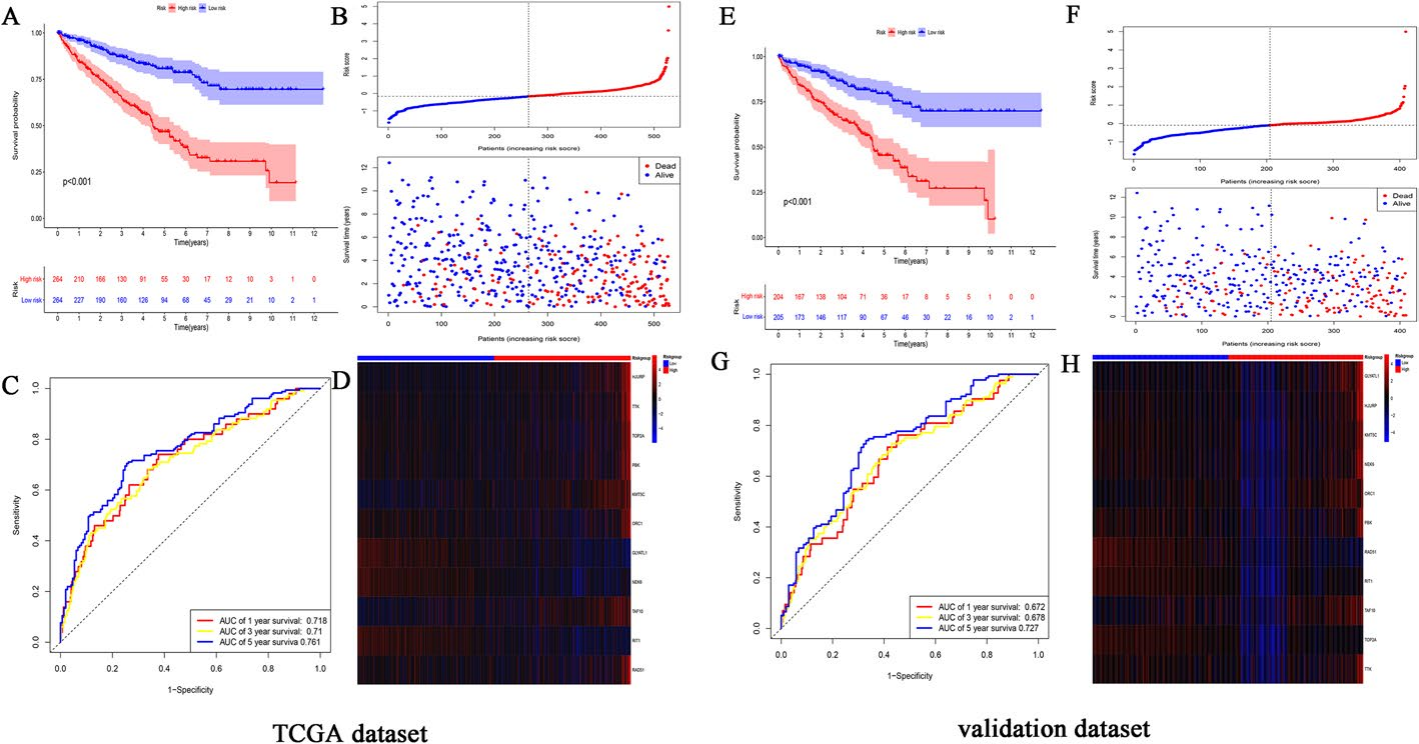
Construction of the prognostic CR-based model in TCGA dataset and validation dataset. **A** Kaplan–Meier survival analysis of TCGA patients between high-risk groups and low-risk groups; **B** Time-independent receiver operating characteristic (ROC) analysis of risk scores predicting the overall survival; **C** Distribution of survival status based on the median risk score; **D** Heatmap showed the differences of 11 chromatin regulators between high and low-risk patients. **E** Kaplan–Meier survival analysis of KIRC patients between high-risk groups and low-risk groups in validation set; **F** Time-independent receiver operating characteristic (ROC) analysis of risk scores predicting the overall survival; **G** Distribution of survival status based on the median risk score; **H** Heatmap showed the differences of 11 chromatin regulators between high and low-risk patients

In this study, we systematically analyzed the function and prognostic value of CRs in KIRC by several effective statistical methods. The KIRC data were exported from the TCGA, including 541 tumor samples and 72 normal samples. The limma R package was utilized to pick out the differentially expressed CRs. A total of 853 CRs were identified. With the standard thresholds *|logFC*|> 1 and *FDR* < 0.05, we identified 127 differentially expressed CRs in the renal clear cell carcinoma tissues compared with the normal tissues. The layout of the top 25 CRs sorted by FDR value is shown in Fig. 1. By performing univariate Cox regression analysis, 20 prognosis‐associated CRs remained (Fig. 2). Then, the final 11 candidate CRs associated with prognosis were analyzed by LASSO Cox regression. (Table 1). Use these eleven CRs to build the final prognostic risk model. Calculate the risk score of each patient according to the formula we introduced as follows: Risk score = (0.0384 * Exp HJURP) + (0.0217 * Exp TTK) + (0.0068 * Exp TOP2A) + (0.0114 * Exp PBK) + (0.0254 * Exp KMT5C) + (0.0059 * Exp ORC1) + (− 0.0040 * Exp GLYATL1) + (− 0.0039 * Exp NEK6) + (0.1874 * Exp TAF10) + (− 0.0022 * Exp RIT1) + (− 0.0585 * Exp RAD51). Subsequently, we classified all patients into a high or low risk groups based on the median risk score. The results showed that the overall survival (OS) of the low-risk group was significantly better (*P* < 0.05) (Fig. 3A). To further assess the prognostic utility, we performed an ROC curve analysis to evaluate the diagnostic value of the risk model. The results showed that the model's accuracy in predicting the prognosis at 1, 3, and 5 years was 0.718, 0.71, and 0.761, respectively (Fig. 3B). The expression heat map of the high and low risk group, the survival status of the patient, and the risk score of the signature consisting of eleven CRs are shown in Fig. 3C and D. To study the model's generalizability, we randomly selected 70% of patients from this dataset plus GSE29609 patients as a validation dataset. OS in the low-risk group increased significantly in the validation dataset (Fig. 3E–H). Our results indicate that the risk model has better specificity and sensitivity.

**Table 1:** 11CRs and coefficient

Chromatin regulators	Coefficient
HJURP	0.038401
TTK	0.021732
TOP2A	0.00688
PBK	0.011418
KMT5C	0.025406
ORC1	0.005983
GLYATL1	− 0.0041
NEK6	− 0.00395
TAF10	0.187492
RIT1	− 0.00226
RAD51	− 0.05851

### A CRs risk model for predicting survival

**Fig. 4:**
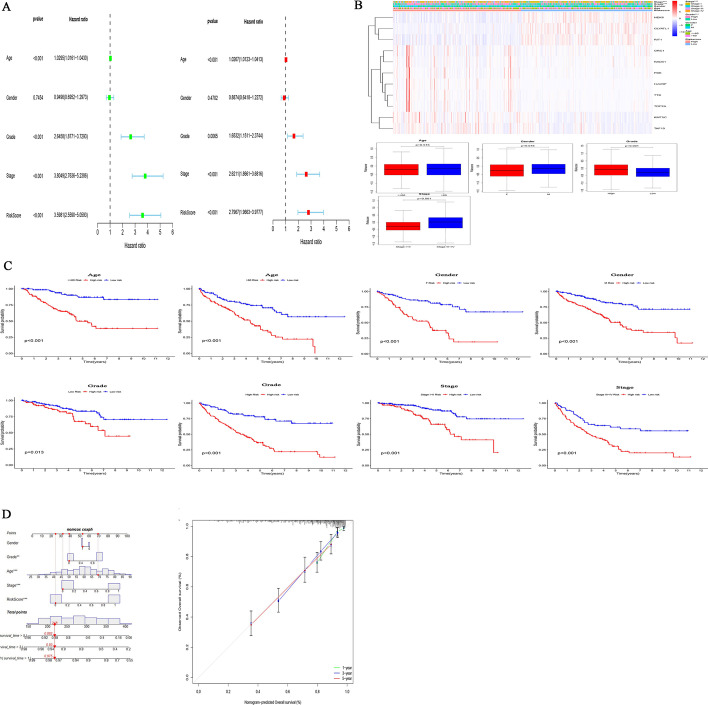
The CRs-based risk model was an independent prognostic factor for KIRC patients. **A** The correlations between the risk score for OS and clinicopathological factors by univariate Cox regression and multivariate Cox regression analysis; **B** Correlation between CRs-based risk model and clinical characteristics. *F* female, *M* male; **C** Kaplan–Meier curves of OS differences stratified by age, gender, grade, TNM stage between the high-risk groups and low-risk groups. *F* female, *M* male; **D** Nomogram for first KIRC patient predicting 1, 3 or 5 year OS, the calibration plots for predicting 1, 3 or 5 year OS

We conducted a univariate and multivariate Cox proportional hazards analysis to clarify the impact of the risk score on prognosis. In these analyses, high risk score, as well as patient age, tumor grade and stage, indicated poor prognosis (*P* < 0.05) (Fig. 4A). These results demonstrated that CR-based signature was an independent prognostic indicator for KIRC patients.

### Association between CRs-based prognostic models and clinical features

The Chi-square test or Fisher's test was used to test whether the model was involved in the progression of KIRC. The results showed significant differences in patient gender, tumor grade, and tumor stage between different risk groups, but no significant differences in age (Fig. 4B). Subgroup analysis was further performed on all patients. The results (Fig. 4C) showed that patients in the low-risk group had more prolonged survival in all subgroups, such as whether they were older than 60 years old, male or female, tumor differentiation, and tumor stage.

### Construction of a nomogram for KIRC patients

In order to develop a simple way for clinical prediction of patient OS, we integrated all clinical characteristics to build a nomogram for predictive model (Fig. 4D). The nomogram is an effective way to show the Cox regression results. We draw a vertical line to determine the expression of the gender, and select the factor score from the normalized 0–100. Use the same method to get the score of the remaining characteristics, and add all the scores. The sum of these scores is on the total score axis, and a downward line is drawn to the survival axis to determine the probability of survival for 1, 3, and 5 years. The C-index of the nomogram is 0.765, which shows that the nomogram can help relevant practitioners make clinical decisions for patients with KIRC. The results of the calibration curve show that the predicted value of the patient is consistent with the actual survival time (Fig. 4D).

### Biological function analysis of differentially expressed CRs and GSEA

**Fig. 5:**
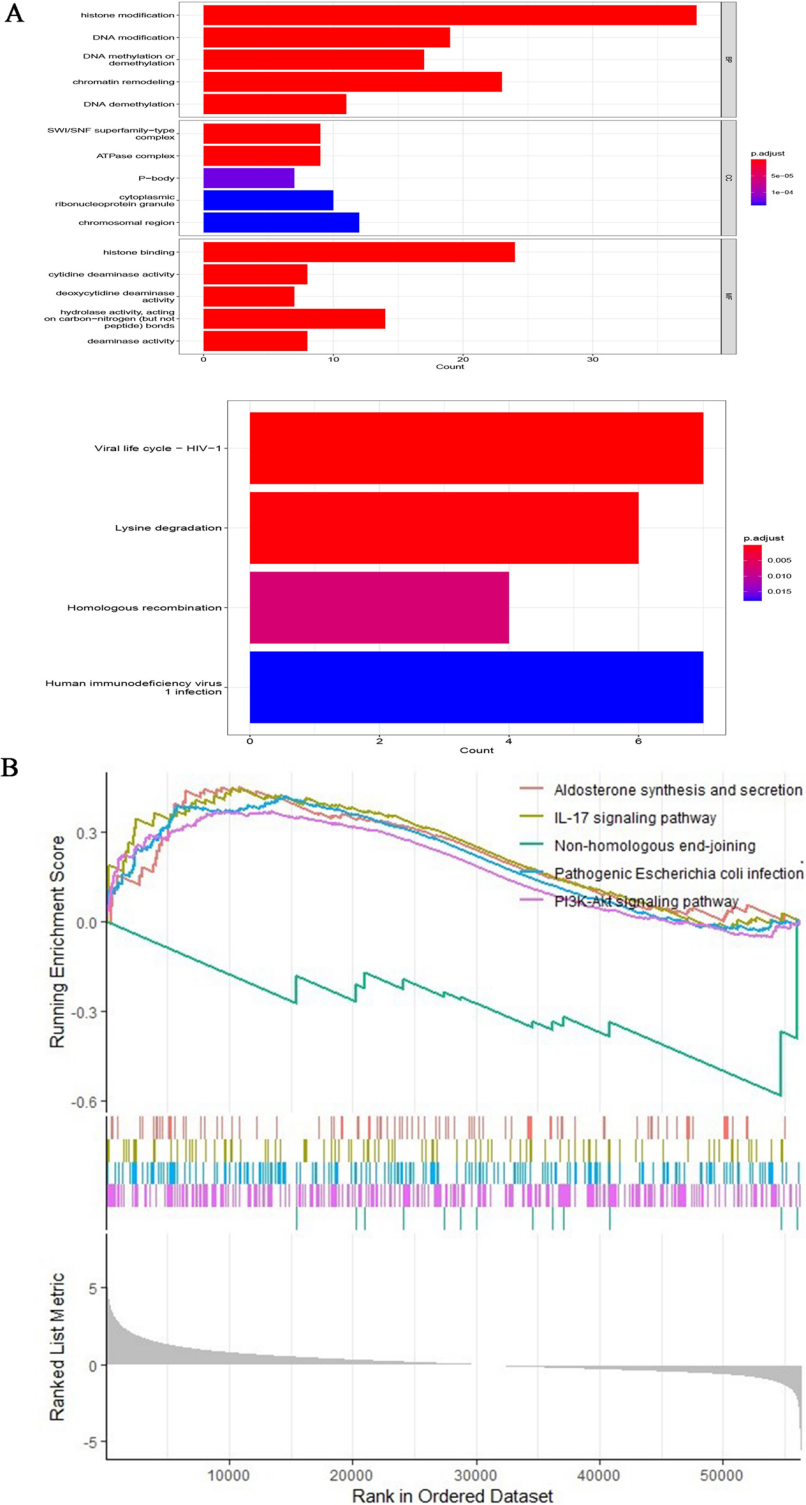
Bioinformatics analysis. **A** GO analysis and KEGG analysis; **B** GSEA analysis

All differentially expressed CRs were analyzed to explore their functions and mechanisms further. The most highly enriched BP associated GO term were histone modification and chromatin remodeling (Fig. 5A). In the CC analysis, the CRs significantly enriched in cytoplasmic ribonucleoprotein granule and chromosomal region (Fig. 5A). For MF terms, histone binding and hydrolase activity, acting on carbon–nitrogen (but not peptide) bonds were enriched by most CRs (Fig. 5A). Besides, the results of KEGG pathway analysis showed that Lysine degradation and Homologous recombination were significantly enriched (Fig. 5A). GSEA analysis helps us further understand the molecular mechanisms involved in all genes in the high and low risk groups distinguished by the CRs prognostic model. The results showed that Aldosterone synthesis and secretion, IL-17 signaling pathway, Pathogenic Escherichia coli infection and PI3K-Akt signaling pathway were mainly enriched in the high-risk group. In contrast, those in the low-risk group were primarily enriched in Non-homologous end-joining (Fig. 5B).

### Analysis of immune infiltration level based on CRs model

**Fig. 6:**
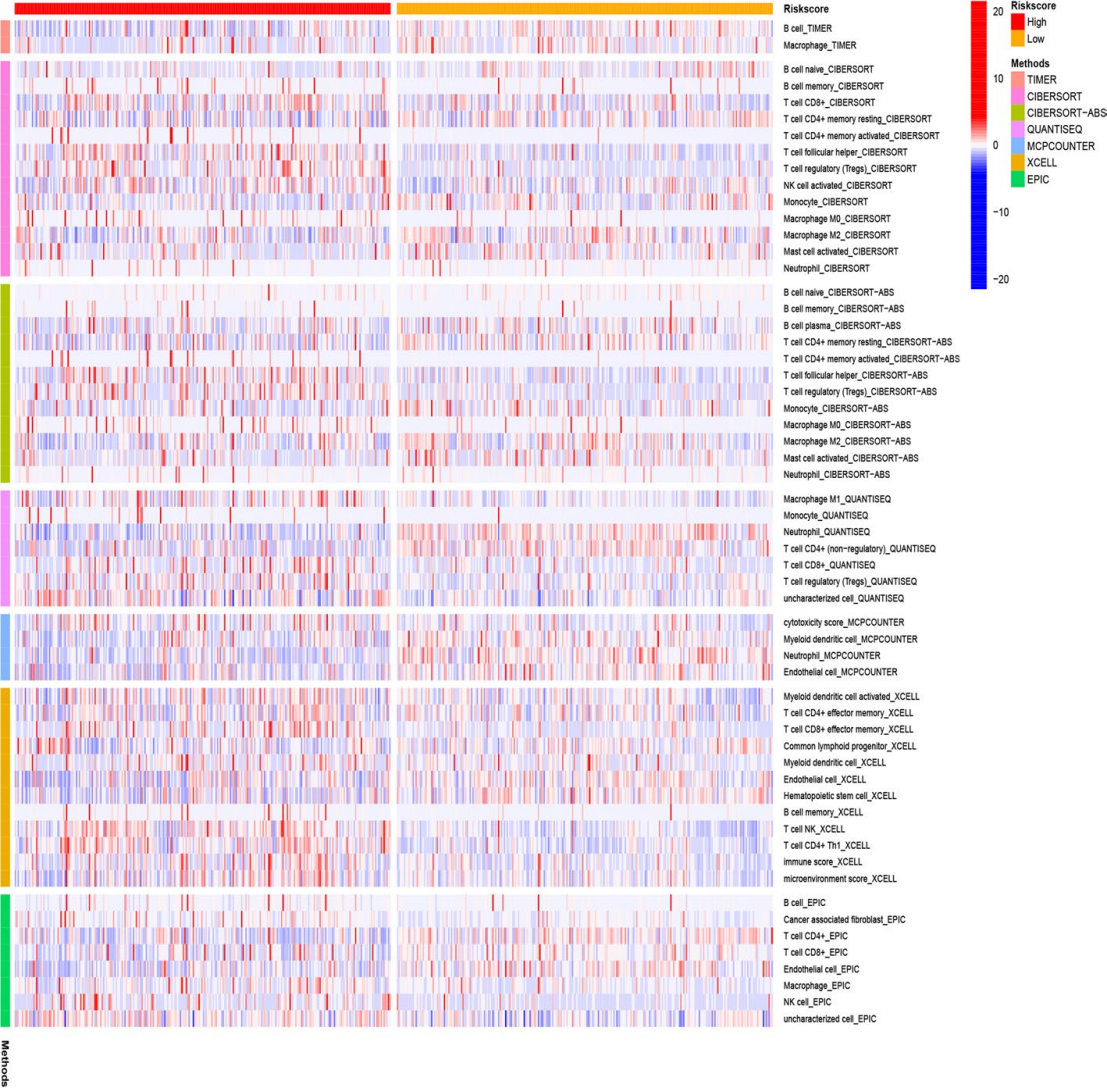
Immune cells infiltration in two risk groups

**Fig. 7:**
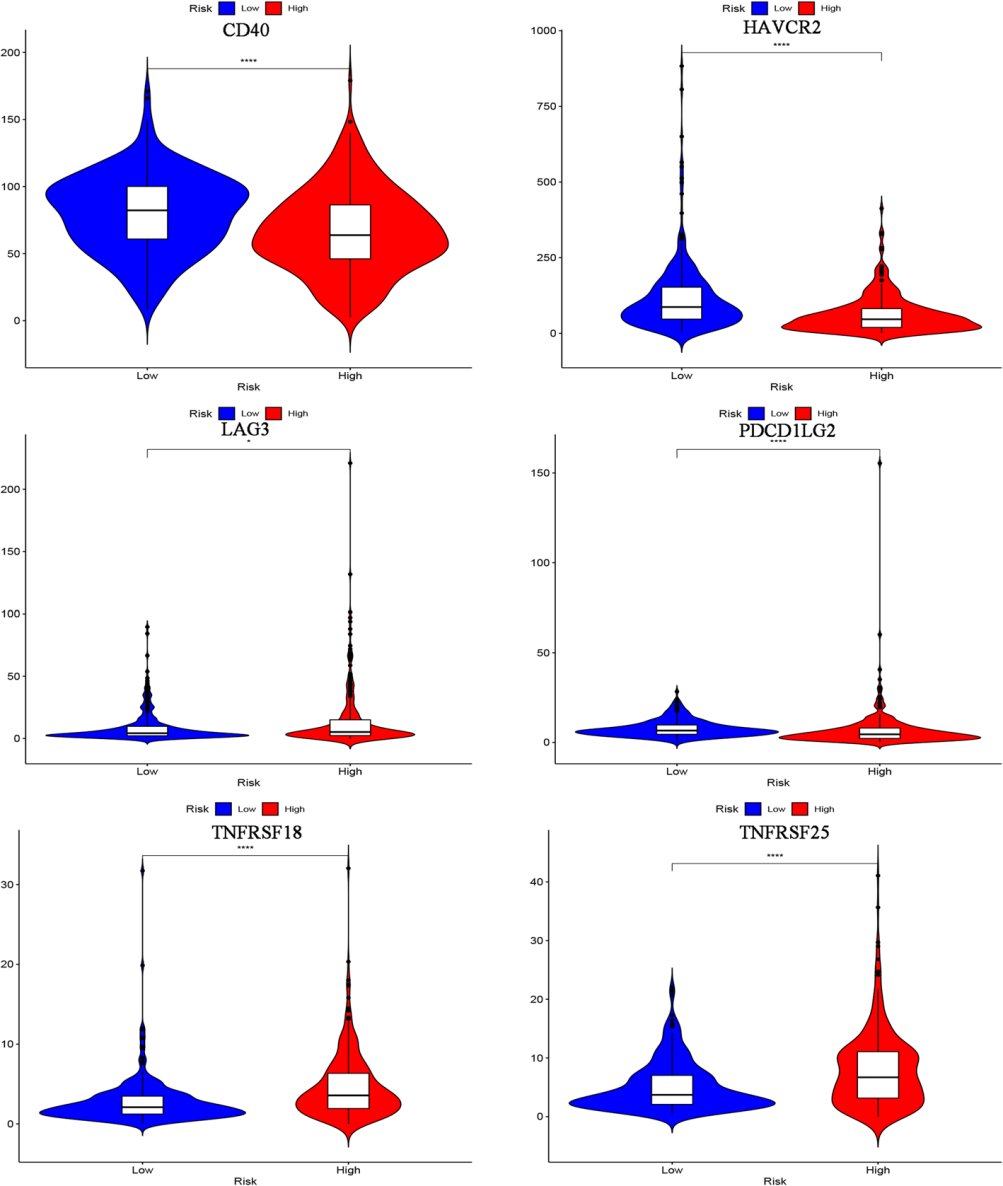
The relationship between prognostic model and immune checkpoints

The heat map (Fig. 6) shows the analysis results of the high and low risk groups by the TIMER, CIBERSORT, CIBERSORT-ABS, XCELL, QUANTISEQ, EPIC and MCP-counter algorithms. These calculation results allow us to intuitively understand that the gene expression of high and low risk groups in different types of immune cells is significantly different, which may be one of the reasons for the significant difference in prognosis. Immune checkpoints are a class of immunosuppressive molecules. During the occurrence and development of tumors, immune checkpoints have become one of the main reasons for immune tolerance. To this end, we also investigated the expression between different risk groups and immune checkpoints. The results showed significant differences in the expressions of CD40, HAVCR2, LAG3, PDCD1LG2, TNFRSF18 and TNFRSF25 between the two groups of patients. In the high-risk group, the expression of tumor necrosis factor superfamily receptor/superfamily (TNFSF/TNFRSF) was high (Fig. 7). Finally, we used the TIMER database to clarify the relationship between the 11 CRs that comprise the prognostic model and immune cells. HJURP, NEK6, RAD51, RIT1, TOP2A, and TTK were positively correlated with immune cells such as B cells, CD8+ T cells, CD4+ T cells, macrophages, neutrophils, and dendritic cells. PBK was positively correlated with B cells, CD8+ T cells, macrophages, neutrophils, and dendritic cells. ORC1 (SLC25A15) is positively associated with immune cells such as B cells, macrophages, neutrophils, and dendritic cells. GLYATL1 is positively correlated with B cells and CD8+ T cells. TAF10 negatively correlated with CD8+ T cells, CD4+ T cells, macrophages and neutrophils. KMT5C (SUV420H2) was positively correlated with CD4+ T cells, and negatively correlated with B cells and CD8+ T cells (Additional file 3: Fig. S1, Additional file 4: Fig. S2 and Additional file 5: Fig. S3).

### Drug sensitivity analysis of KIRC Patients

**Fig. 8:**
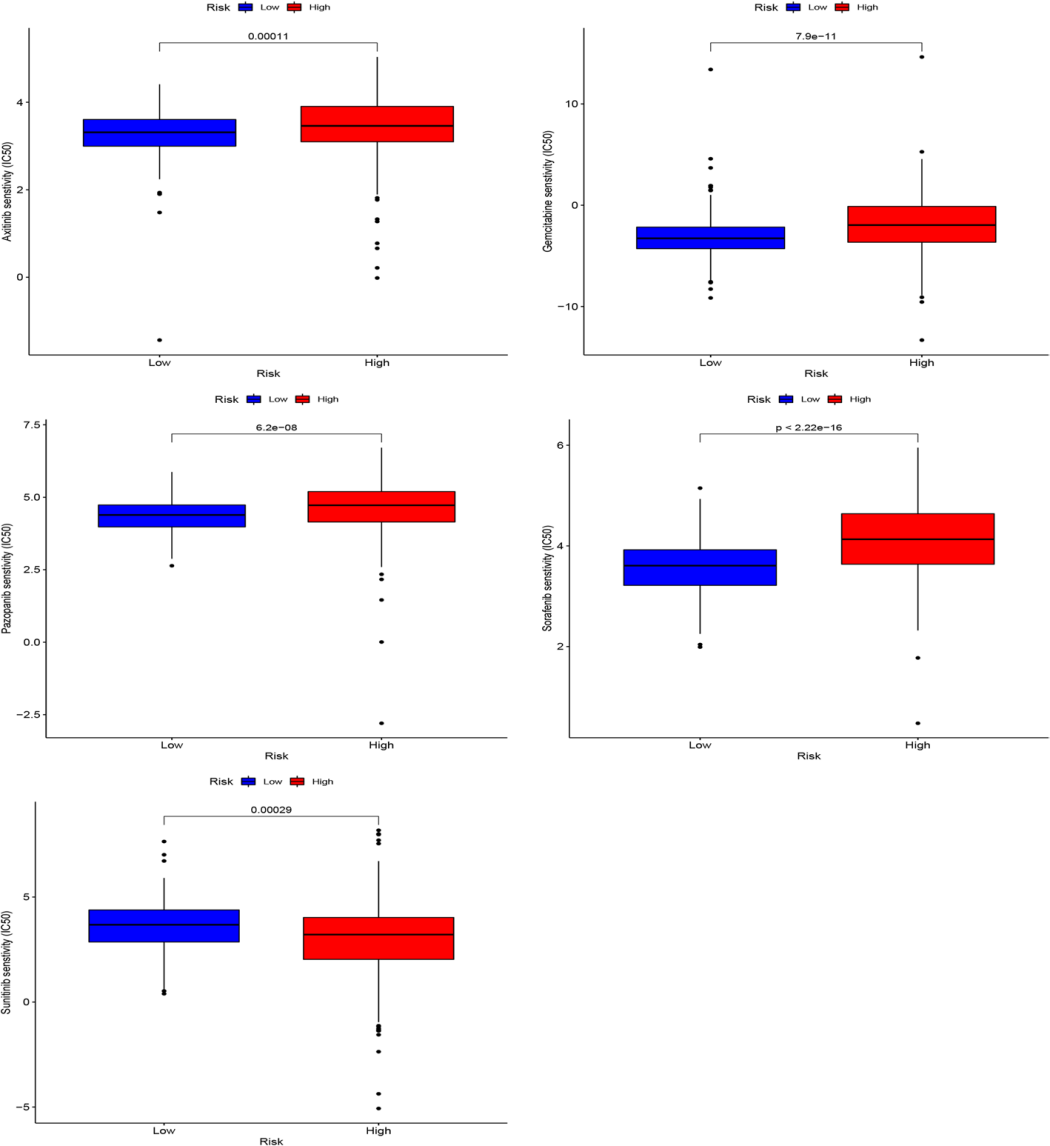
Drug sensitivity analysis

By analyzing the commonly used drugs in KIRC patients through the GDSC database, we found that drugs such as Axitinib, Pazopanib, Sorafenib and Gemcitabine have higher IC50 values in patients in the high-risk group than those in the low-risk group, indicating that the patients in the low-risk group are more sensitive to these drugs. However, the IC50 value of Sunitinib was lower than that of patients in the low-risk group, suggesting that patients in the high-risk group may be more sensitive to Sunitinib (Fig. 8).

## Discussion

With the in-depth study of chromatin regulators by researchers from various countries, we found they are essential participants in malignant tumors. Abnormal CRs functions and dysregulated expression may promote the occurrence and progression of tumors, but the process of most CRs in tumors is still unclear [26–30]. Therefore, understanding the interaction between CRs and tumors may provide new clinical treatment strategies for patients. In this study, 127 differentially expressed CRs at the mRNA expression level were identified using KIRC data from the TCGA database. Furthermore, univariate Cox regression and Lasso Cox regression analysis identified eleven key CRs, which were used to establish the prognostic model. We also performed survival analysis, ROC analysis, univariate COX and multivariate COX analysis on the high and low risk groups distinguished by the prognostic model. We determined that it can effectively indicate the survival risk of KIRC patients and is an independent predictor of prognosis. The above results have been validated by 70% randomization of this dataset.

GO enrichment analysis showed that these CRs were mainly associated with epigenetic processes, such as histone modification, chromatin remodeling, cytoplasmic ribonucleoprotein granule and histone binding. Previous studies have shown that Methyltransferase-like 14 (METTL14) is involved in the tumorigenesis of various malignant tumors. The down-regulated METTL14 in renal clear cell carcinoma can accumulate bromodomain PHD finger transcription factor (BPTF) and cause distant lung metastasis through chromatin remodeling [31]. It has also been found that tumor driver genes IDH1/2, JARID1C/KDM5C and UTX/KDM6A can regulate histone demethylation and thus affect cancer metabolism and tumor progression [32].

When conducting KEGG pathway analysis, we found that 127CRs were significantly enriched in lysine degradation and homologous recombination. GSEA analysis showed that the molecular mechanisms involved in CRs were mainly Aldosterone synthesis and secretion, IL-17 signaling pathway, Pathogenic *Escherichia coli* infection and PI3K-Akt signaling pathway. Many previous studies have demonstrated that these molecular pathways and mechanisms are closely related to the occurrence of cancer and tumor cell metabolism [33–35]. These findings may be helpful to develop biomarkers for diagnostic or prognostic of KIRC patients.

Holliday junction recognition protein (HJURP), with histone binding activity and the same protein binding activity. Some researchers found that HJURP is a potential independent prognostic marker of ccRCC and can play an important role in the tumor microenvironment by regulating immune cell infiltration [36]. In glioblastoma (GBM), HJURP is often overexpressed, and the knockdown of HJURP disrupts the colony-forming ability of GBM cells and increases their radio sensitivity [37]. TTK protein kinase (TTK), encodes a dual-specificity protein kinase with the ability to phosphorylate tyrosine, serine, and threonine and is associated with cell proliferation. Previous studies have found that the expression level of TTK is significantly correlated with clinical characteristics such as the T stage and N stage in ccRCC patients. Knockdown of TTK inhibited cell proliferation and invasion in 2 ccRCC cells, HTB-47 and CRL-1932 cells. Furthermore, TTK contributes to tumor growth and metastasis in mouse ccRCC [38]. DNA topoisomerase II alpha (TOP2A) is a DNA topoisomerase involved in processes such as chromosome condensation, chromatid separation, and DNA transcription and replication, and is the target of several anticancer drugs [39, 40]. PDZ-binding kinase (PDK) is a dual-specificity mitogen-activated protein kinase kinase (MAPKK) family-related serine/threonine protein kinase. TOPK/PBK (T-LAK cell-derived protein kinase) is a serine/threonine kinase that is highly expressed in a variety of human tumors and is associated with poor prognosis in various human malignancies. In KIRC, Ser32 was found to be a novel phosphorylation site on TOPK that can be activated by ERK2. Combining a TOPK inhibitor with sorafenib promotes apoptosis in sorafenib-resistant RCC [41]. Lysine methyltransferase 5C (KMT5C) can initiate histone binding activity and histone methyltransferase activity. Deletion of KMT5C in non-small cell lung cancer promotes resistance to EGFR inhibitors through the LINC01510/MET axis, which could lead to improved mechanistic insights into NSCLC therapy [42]. The origin recognition complex subunit 1 (ORC1) is a highly conserved six-subunit protein complex essential for initiating DNA replication in eukaryotic cells. It has been reported that ORC1 may become a new prognostic marker for glioma by activating the ERK/JNK signaling pathway [43]. Glycine-*N*-acyltransferase like 1 (GLYATL1) is mainly involved in glutamine metabolism and can enable glutamine *N*-acyltransferase activity. Pseudogene PLGLA can inhibit the proliferation and division of liver cancer cells by regulating the miR-324-3p/GLYATL1 axis [44]. The NIMA-related kinase 6 (NEK6) encoded by this gene is a kinase required for metaphase progression.FAM13A-AS1, a less-studied lncRNA, is upregulated in RCC patients and promotes tumorigenesis by competitively binding to miR-141-3p and upregulating NEK6 expression [45]. TATA-box binding protein associated factor 10 (TAF10) may be involved in basal transcription, act as a coactivator, play a role in promoter recognition or modify general transcription factors (GTFs) to facilitate complex assembly and transcription initiation [46]. Ras like without CAAX 1 (RIT1) encodes a member of the Ras-associated GTPases subfamily involved in regulating the p38 MAPK signaling pathway associated with cellular stress. Excessive activation of RAS/MAPK signaling is commonly observed in hepatocellular carcinoma (HCC), and it has been found that RIT1 induces angiogenesis through the MEK/ERK/EIF4E/HIF1-α/VEGFA axis. Furthermore, RIT1 increases the phosphorylation of p38 MAPK and AKT to promote cell survival under reactive oxygen species stress [47]. The protein encoded by RAD51 recombinase (RAD51) is a member of the RAD51 protein family. This protein interacts with BRCA1 and BRCA2, which may be necessary in the cellular response to DNA damage. Loss of these controls following BRCA2 inactivation may be a key event leading to genomic instability and tumorigenesis [48].

We also explored the relationship between different risk groups and immune checkpoints. In the high-risk group, the expression of tumor necrosis factor superfamily receptor/superfamily (TNFSF/TNFRSF) [49, 50] was higher than that in the low-risk group. At the same time, the expression of CD40, HAVCR2, LAG3, and PDCD1LG2 is more in the low-risk group. This shows that there may be immunosuppression in both high- and low-risk groups, and more targeted immune checkpoint inhibitors should be developed or used in response to the difference in the expression of the immune checkpoints between the two groups to prolong survival and reduce the economic pressure of patients. Through the analysis of the TIMER database, we found that 11CRs are closely related to tumor immune cell infiltration, which indicates that 11 CRs may also be involved in the process of immune response, which is worthy of our further study. The final drug sensitivity results show that the high-risk group may be better with Sunitinib, while the low-risk group may be better with Axitinib, Pazopanib, Sorafenib and Gemcitabine.

Our research still has some limitations. First, our original data comes from an online database. However, the implementation of mutual verification illustrates the feasibility of this risk model. But then, we may still need to add more data samples for further proof. Second, establishing a prognostic model involves a lot of statistics and the application of R language, so it is necessary to master the relevant knowledge. Overall, our prognostic risk model is more conducive to popularising clinical applications than the high cost of next-generation sequencing.

## Conclusions

In summary, the prognostic risk of patients with renal clear cell carcinoma can be assessed using this chromatin regulators associated prognostic model. At the same time, these CRs in the model are most likely related to the progression of KIRC. Therefore, our results can bring new ideas for targeted therapy of renal clear cell carcinoma, and help doctors take corresponding measures for patients with different risks in advance.

## Supplementary Information

**Additional file 1.** List of 870 chromatin regulators.

**Additional file 2.** List of chromatin regulators screened from the TCGA dataset.

**Additional file 3: Fig. S1.** TIMER database result.

**Additional file 4: Fig. S2.** TIMER database result.

**Additional file 5: Fig. S3.** TIMER database result.

## Data Availability

The datasets analyzed for this study can be found in the TCGA (https://portal.gdc.cancer.gov/).

## Abbreviations

KIRC: Renal clear cell carcinoma
CRs: Chromatin regulators
DPF3a: The short isoform of DPF3
ccRCC: Clear cell renal cell carcinoma
TCGA: The Cancer Genome Atlas
ICGC: The International Cancer Genome Consortium database
OS: Overall survival
C-index: Concordance index
CC: Cellular component
BP: Biological process
MF: Molecular function
GSEA: Gene set enrichment analysis
GDSC: The genomics of drug sensitivity in cancer database
IC50: The half-maximal inhibitory concentration
GO: Gene ontology
CC: Cellular component
BP: Biological process
MF: Molecular function
HJURP: Holliday junction recognition protein
GBM: In glioblastoma
TTK: TTK protein kinase
MAPKK: Mitogen-activated protein kinase kinase
PDK: PDZ-binding kinase
TOP2A: Topoisomerase II alpha
TOPK/PBK: T-LAK cell-derived protein kinase
KMT5C: Lysine methyltransferase 5C
ORC1: The origin recognition complex subunit 1
GLYATL1: Glycine-*N*-acyltransferase like 1
TAF10: TATA-box binding protein associated factor 10
NEK6: NIMA-related kinase 6
GTFs: General transcription factors
RIT1: Ras like without CAAX 1
HCC: Hepatocellular carcinoma
RAD51: The protein encoded by RAD51 recombinase
TNFSF/TNFRSF: The expression of tumor necrosis factor superfamily receptor/superfamily
