# Supplementary figures and images for: Establishing a prognostic model of chromatin modulators and identifying potential drug candidates in renal clear cell patients
Source: BMC Bioinformatics. 2023 Mar 20;24:104. doi: 10.1186/s12859-023-05229-9 (PMC10029171; doi:10.1186/s12859-023-05229-9)

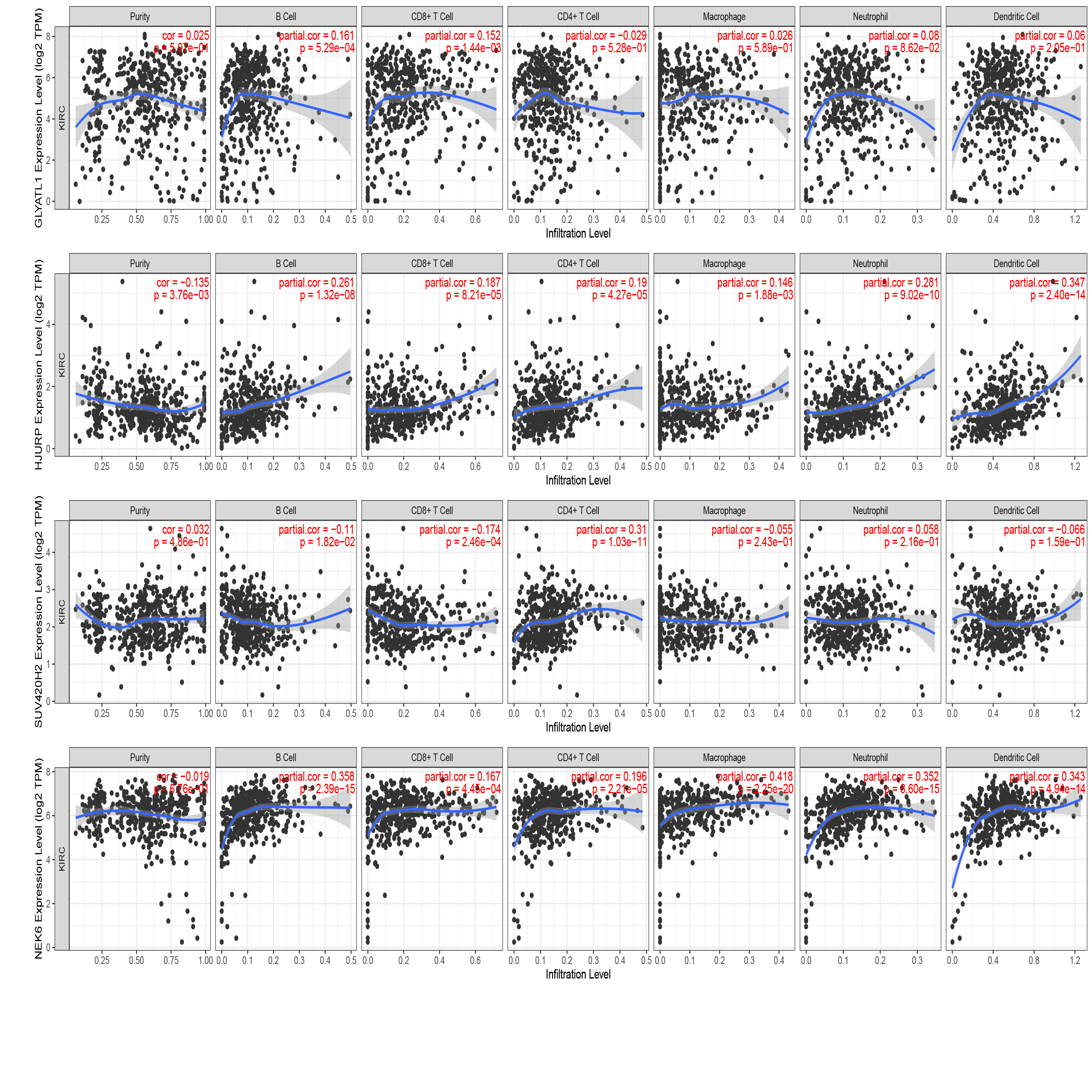

Supplement: Supplementary file 3 — Additional file 3: Fig. S1. TIMER database result. [file 12859_2023_5229_MOESM3_ESM.jpg]

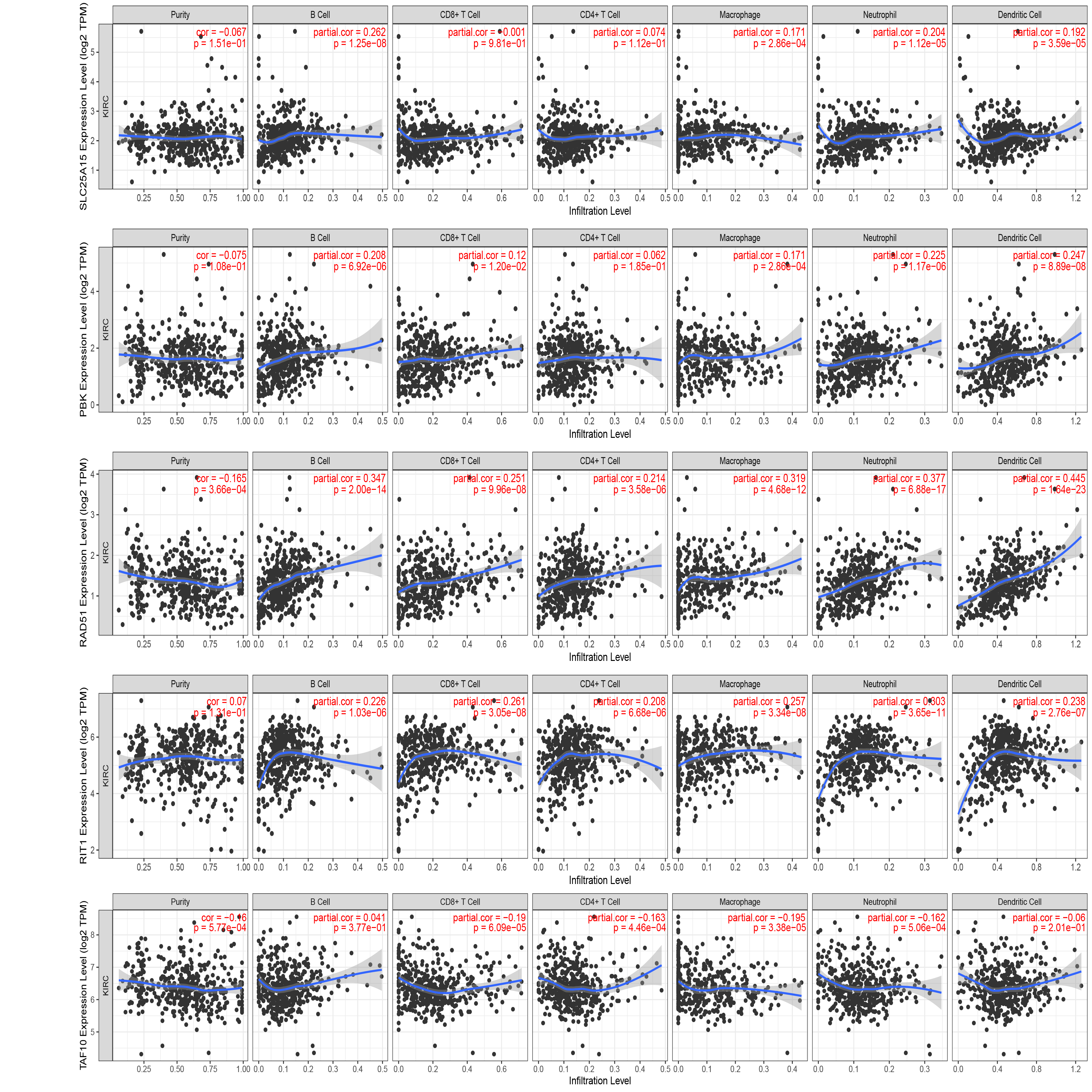

Supplement: Supplementary file 4 — Additional file 4: Fig. S2. TIMER database result. [file 12859_2023_5229_MOESM4_ESM.jpg]

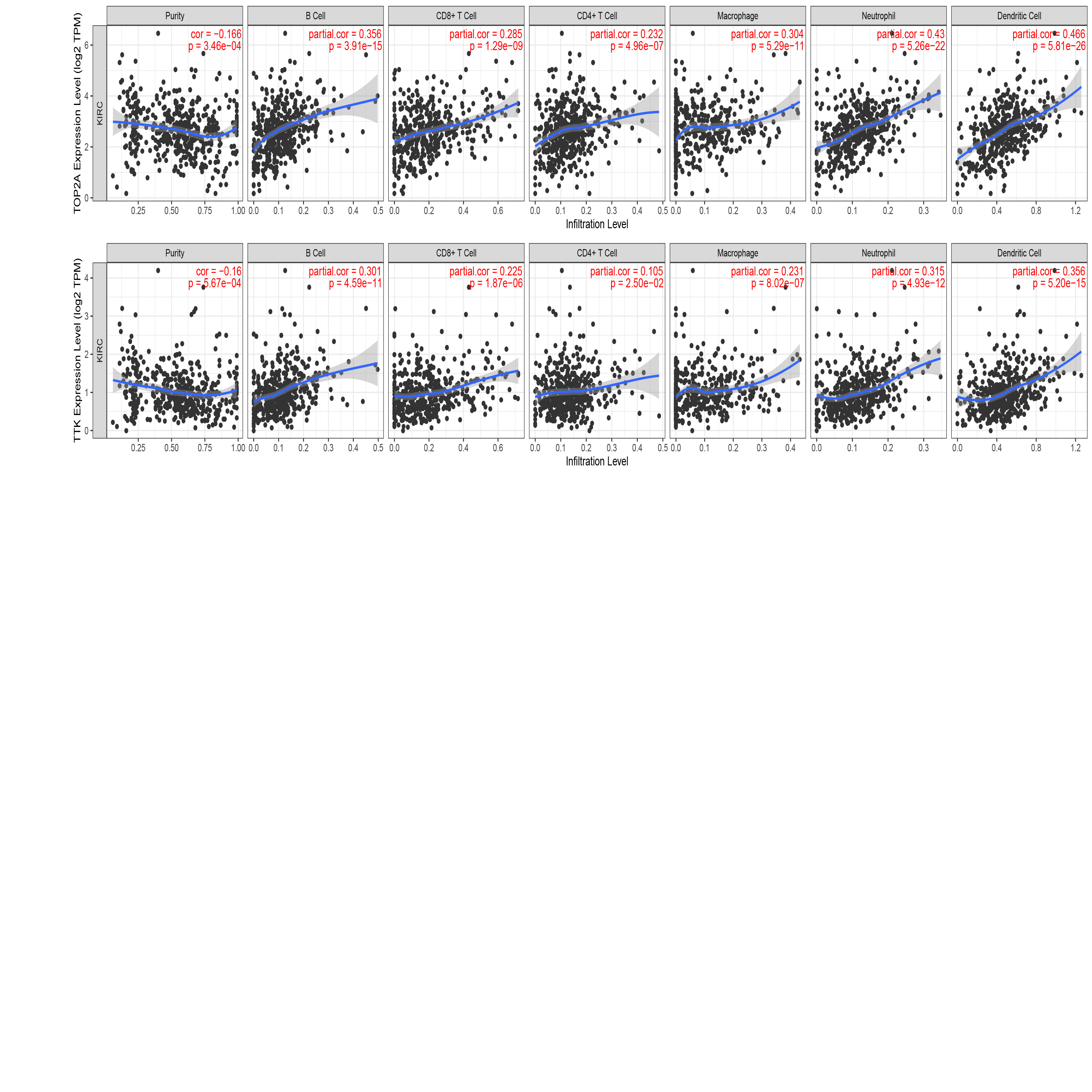

Supplement: Supplementary file 5 — Additional file 5: Fig. S3. TIMER database result. [file 12859_2023_5229_MOESM5_ESM.jpg]
